# A multi-perspective assessment of knowledge, attitudes, and barriers to viral hepatitis care in Ghana

**DOI:** 10.3389/fcimb.2026.1776176

**Published:** 2026-05-13

**Authors:** Dorcas Ohui Owusu, Bright Afriyie Owusu, Augustine Yeboah, Melody Ama Antwi, Enusah Nandonfali, Sampson Agyapong Abakah, Michael Owusu

**Affiliations:** 1 Department of Theoretical and Applied Biology, Kwame Nkrumah University of Science and Technology, Kumasi, Ghana; 2 Kumasi Centre for Collaborative Research in Tropical Medicine, Kwame Nkrumah University of Science and Technology, Kumasi, Ghana; 3 Department of Medical Laboratory Science, University of Energy and Natural Resources, Sunyani, Ghana; 4 Department of Molecular Medicine, Kwame Nkrumah University of Science and Technology, Kumasi, Ghana; 5 Centre for Health Systems Strengthening, Kumasi, Ghana; 6 Department of Medical Diagnostics, Kwame Nkrumah University of Science and Technology, Kumasi, Ghana

**Keywords:** Ghana, health knowledge, healthcare barriers, hepatitis b, hepatitis c, perception, viral hepatitis

## Abstract

Viral hepatitis B and C are a significant public health burden in Ghana, yet elimination efforts, as outlined by the WHO, are hindered by gaps in understanding and access to care. This cross-sectional study aimed to assess the knowledge, misconceptions, and barriers to care from the critical perspectives of patients, caregivers, and healthcare workers (HCWs) in Ghana. We used structured questionnaires to evaluate the awareness, risk perception, understanding of transmission, chronicity, treatment preferences, and recognition of high-risk groups among 449 participants. While general awareness was high, specific knowledge about sources of infection and symptoms was poor among the patient participants (p < 0.05). Less than 50% of patient participants were aware of their HCV status. About 4-20% of all the study participants believed the disease is caused by ageing, having malaria, eating oily foods, witchcraft and curses, or engaging in laborious work. Disparity existed in serostatus awareness (HCWs: 91.2%; patients and caregivers: 56%) and personal risk perception. Major barriers included cost (cited by 75%), distance, and unpleasant interactions between patient-care providers. Most study participants correctly identified clinical risk groups. However, 16% of community participants failed to recognise multiple sexual partners as a risk factor. The findings reveal a significant gap between the aetiology of viral hepatitis disease and community understandings of the disease, driven by misconceptions and structural barriers. To achieve elimination targets as outlined by the World Health Organisation, public health strategies must integrate cultural beliefs into education, destigmatisation, and health system strengthening to improve access to prevention, testing, and treatment services.

## 1 Introduction

Viral hepatitis, mostly caused by the hepatitis B (HBV) and C (HCV) viruses, is a global public health challenge, with approximately 296 million (3·8%) people living with chronic HBV infection and 58 million (0·8%) people living with chronic hepatitis C virus (HCV) infection (Cui et al., 2023). This prevalence has contributed to over one million deaths annually, as infected persons suffer liver cirrhosis and hepatocellular carcinoma (Alberts et al., 2022). The burden of this silent epidemic has a significant impact on low and middle-income countries as it contributes to about 50% of liver disease across the Sub-Saharan Africa region (Layden et al., 2015; Sonderup et al., 2017; Spearman et al., 2017; Alberts et al., 2022; World Health Organisation, 2025). In Ghana, the estimated hepatitis B and C seroprevalence is 11.4% and 5.32%, respectively, indicating millions of Ghanaians are living with a potentially life-threatening condition (Nartey et al., 2022; Nartey et al., 2023; Fosu et al., 2025). Despite the availability of an effective vaccine for HBV and curative direct-acting antivirals for HCV, the World Health Organisation’s goal of eliminating hepatitis by 2030 remains a challenge to achieve due to systemic and community-level barriers (Tantuoyir et al., 2024). In addition to resource limitations, a complex interplay of knowledge deficits, deep-seated misconceptions, and multifaceted barriers to care remains to be fully understood (Freeland et al., 2024).

The fundamental setback in controlling viral hepatitis in Ghana may lie in knowledge gaps of the disease, including experiences and health-seeking behaviours of infected persons. Even though clinical guidelines exist, their implementation may be hindered by the attitude of persons with the disease. Importantly, the levels of basic awareness and accurate knowledge regarding viral hepatitis types, their modes of transmission, disease condition, and co-infection potentials among patients, their caregivers, and even HWCs are insufficiently documented. These knowledge gaps are often filled with misconceptions along with cultural beliefs about disease, overshadowing biomedical facts, and symptoms may be attributed to other causes, leading to delays in testing and treatment.

Therefore, this study aimed to generate comprehensive, actionable evidence to inform a more effective national response to viral hepatitis. The primary objective is to conduct a multi-perspective assessment of knowledge, attitudes, and practices regarding viral hepatitis in Ghana. The research aimed to assess the level of awareness, basic knowledge, and personal perceptions of the potential for co-infection with viral hepatitis types among patients, caregivers, and HWCs: specifically identified prevalent misconceptions and knowledge gaps regarding viral hepatitis, and evaluated attitudes, practices, and barriers to prevention and treatment. Additionally, determine the perspectives of the studied groups in recognising high-risk groups. The findings provide an evidence base for designing targeted interventions, health education campaigns, and policies that address the real barriers to achieving viral hepatitis elimination in Ghana.

## 2 Materials and methods

### 2.1 Study design and setting

**Figure 1:**
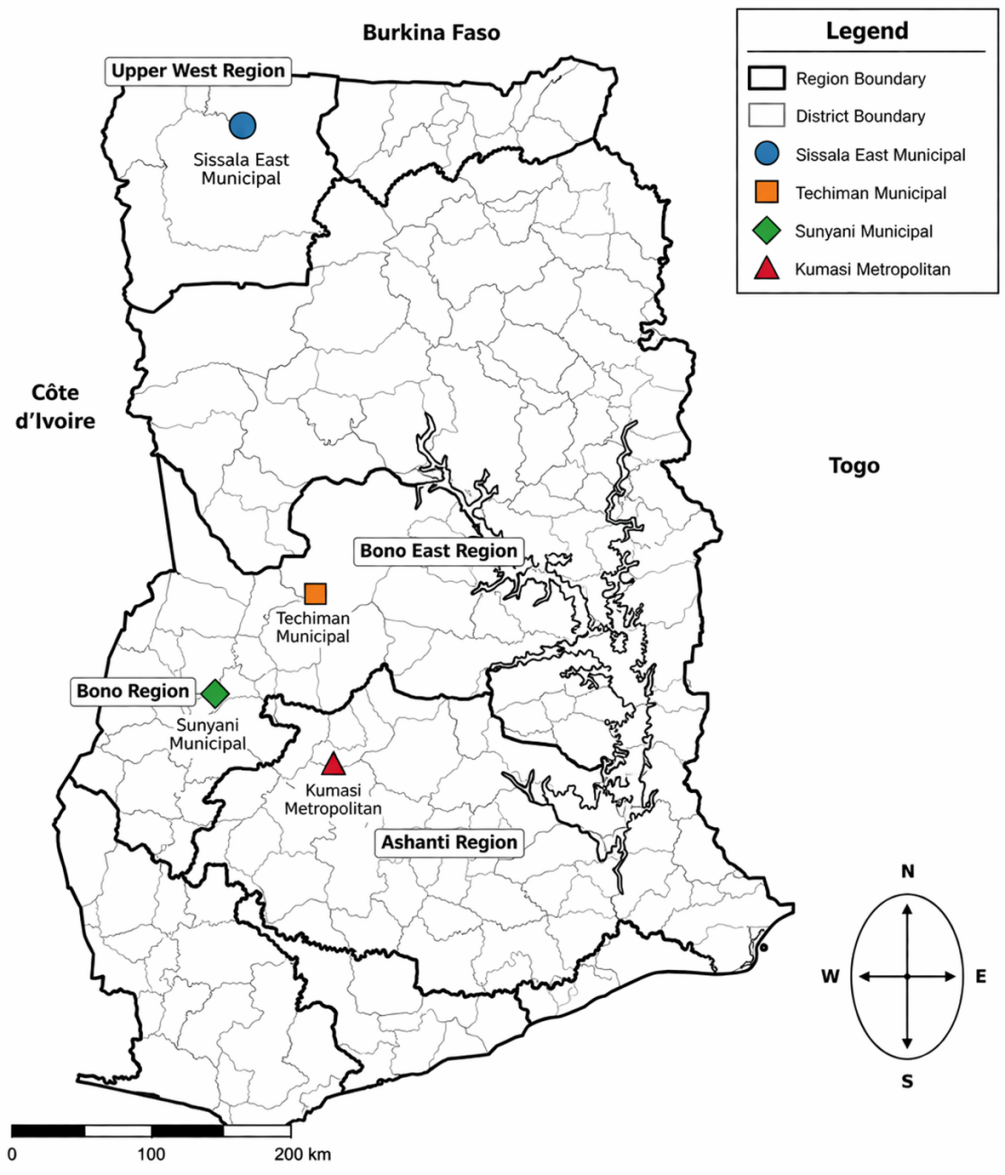
A map of Ghana with selected areas of study.

A descriptive, cross-sectional study was conducted using a well-structured questionnaire to comprehensively assess the perspectives of multiple stakeholder groups. The study was conducted in the outpatient departments and medical wards of selected healthcare facilities in Ghana to ensure diversity. Sites included health centres, district and Regional hospitals in the Northern Region, Bono East Region, and Ashanti Region, as presented in **Figure 1**. This approach captured a wide range of community-level care, extending to referral centres selected to provide a mix of urban and peri-urban patient populations. Data collection occurred over six months.

### 2.2 Study population and sampling

The target populations were three categories of persons: already diagnosed viral hepatitis infected patients (aged 18 years and above) attending general medical clinics, caregivers accompanying such patients to the facilities, and Healthcare Workers (HCWs), including doctors, nurses, midwives, and healthcare assistants, actively involved in patient care in the selected facilities. A purposive sampling approach was employed to ensure representation from key departments among HWCs. For patients and caregivers, a systematic random sampling method was employed, approaching every fifth individual who met the inclusion criteria. A sample size of 423 study participants was estimated using a single proportion, assuming a 50% prevalence of adequate hepatitis knowledge to maximise variability, a 5% margin of error, a 95% confidence level, and an additional 10% allowance for non-response.

Data were collected over a six-month period using a comprehensive, structured questionnaire specifically developed for this study. It was developed and validated in accordance with the study objectives. The study questionnaire was developed following a review of relevant literature and the validation of previously used instruments in hepatitis knowledge and awareness studies, as well as the WHO hepatitis knowledge surveys. The content validity was assessed by experts in public health and infectious diseases for relevance and clarity. The questionnaire was pretested among individuals with characteristics similar to those of the study participants. These persons were not included in the main study. Validity and reliability were evaluated based on participants’ understanding and interpretation of the questions. Necessary revisions were made before final administration.

The questionnaire comprised five main sections, including (i) Socio-demographic Data; (ii) Awareness, Knowledge, and Risk Perception using a 5-point Likert scale; (iii) Misconceptions and Understanding of Disease aetiology; (iv) Attitudes, Practices, and Barriers; and (v) knowledge on high-risk groups.

### 2.3 Data collection procedure

Ethical approval was obtained from the University of Energy and Natural Resources, Sunyani Committee for Human Research and Ethics (CHRE/CA/AP/001/023). Trained research assistants administered the questionnaires. Written informed consent was secured from all participants. Questionnaires were completed through interviewer-administered interviews with patients and caregivers to accommodate varying literacy levels. Healthcare workers were given the option to self-administer the questionnaires to reduce the time required for engagement. All responses were anonymised.

### 2.4 Data analysis

Data were entered into KoboToolbox and analysed using the R statistical package. Descriptive statistics were used to summarise socio-demographic characteristics and responses to knowledge, attitude, and practice items. Likert values ‘Agree’ and ‘Strongly Agree’ are treated as agreement. For items coded as Yes or No, ‘Yes’ is treated as an agreement or a positive response. Chi-square tests were used to examine associations between demographic variables and knowledge levels. Responses from the three participant groups were analysed comparatively.

## 3 Results

A total of 449 participants were enrolled in the study: 62 (13.8%) patients, 57 (12.7%) caregivers, and 330 (73.5%) healthcare workers (HCWs). Among the HCWs, 4.2% were medical doctors, 58.8% were nurses or midwives, and 7% were healthcare assistants. Thirty percent of the health workers in the facilities studied were other health care workers, including cleaners and janitors. The median age of the study participants was 28 years (range: 18-80), and 57.9% were female. Among the patients and caregivers, 53% (63/119) had tertiary education, 24.1% (29/119) had secondary education, 16.8% (20/119) had basic education, and 5.9% (7/119) had no formal education. Forty-five percent (53/119) of the patients and caregivers were employed, as presented in **Table 1**.

**Table 1:** Sociodemographic characteristics of study participants (N = 449).

	Healthcare workers N = 330 (%)	Caregivers N = 57 (%)	Patients N=62 (%)
Age (years)			
Median (min, max)	28 (18, 46)	29 (18, 54)	28 (18, 80)
Sex			
Female	193 (58.5)	37 (64.9)	30 (48.4)
Male	137 (41.5)	20 (35.1)	32 (51.6)
Education level			
No formal education	**-**	2 (3.5)	5 (8.1)
Primary school	2 (0.6)	2 (3.5)	3 (4.8)
JHS	3 (0.9)	5 (8.8)	10 (16.1)
SHS/Tech Sch	10 (3.0)	14 (24.6)	15 (24.2)
Tertiary education	315 (94.8)	34 (59.6)	29 (46.8)
Employment status			
Employed	330 (100)	25 (43.9)	14 (22.6)
Student	–	13 (22.8)	14 (22.6)
Unemployed	-	19 (33.3)	34 (54.8)

JHS, Junior High School; SHS, Senior High School; Tech Sch, Technical School.

### 3.1 Awareness and knowledge about viral hepatitis

Awareness of Hepatitis B was high in all participant categories (96.4%, n = 433), but lower for Hepatitis C (86.4%, n = 388) and Hepatitis D (74.4%, n = 334). Awareness of hepatitis C and D viral infections was significantly lower among patients and caregivers. Seventy-five per cent (n = 339) reported knowing their own hepatitis status, with a higher proportion being HWCs (82.7%, n = 273), as presented in **Supplementary Table 1**.

The perceived causes revealed a mix of accurate and inaccurate beliefs. Blood transfusion was commonly identified as a cause (82.6%), while alcohol intake was also frequently reported (51.0%), with significant differences between the study participant groups. Remarkably, alcohol was less frequently stated as a cause of viral hepatitis by caregivers (40.4%) and patients (35.5%) than by health care workers (55,8%), possibly because the latter bear in mind a possible indirect association of high alcohol intake with viral hepatitis, e.g. through riskier sexual behaviour.

**Figure 2:**
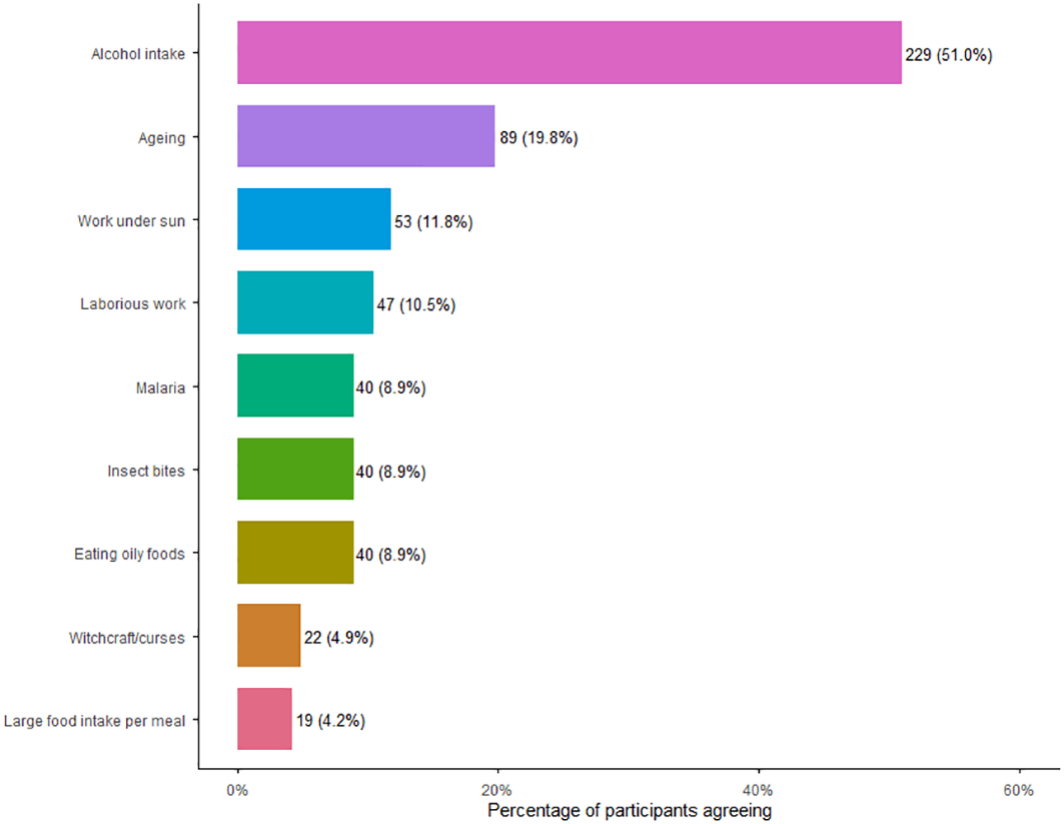
Misconceptions about the causes of viral hepatitis among the study participants.

Misconceptions about the cause of viral hepatitis were present but less common. Belief in witchcraft and curses as a cause differed significantly across groups, with the highest proportion among patients (11.3%), followed by caregivers (7.0%) and HWCs (3.3%) (p = 0.022) Other misconceptions about causes included “working under the sun” (11.8%), engaging in “laborious work” (10.5%), ageing (19.8%) followed by malaria, eating oily foods, and insect bites (**Supplementary Table 1**; **Figure 2**).

Knowledge of signs and symptoms was generally high for several common manifestations, such as fatigue (85.1%), fever (79.5%), jaundice (77.3%), abdominal pain (71.9%), and dark urine (75.1%). However, statistically significant differences were observed among participant groups for certain symptoms. Caregivers reported chills as a symptom of viral hepatitis more often than the other groups (p = 0.001), and patients were less likely to identify dark urine (p < 0.001) as a symptom of viral hepatitis. Recognition of pale stool and anaemia as symptoms of viral hepatitis also differed significantly across groups (both p = 0.002), with patients consistently reporting low agreement (**Supplementary Table 1**).

Knowledge of the mode of disease transmission revealed that a high proportion of study participants correctly identified blood exposure–related risks, such as blood transfusion (93.8%), needle prick (92.2%), sharing personal items (92.7%), and sexual intercourse (91.5%). Misconceptions persisted, with some indicating that insect bites (16.0%) and sharing a common space (23.6%) were modes of transmission.

Identifying high-risk groups was strongest for sexual partners of people with HBV (94.0%) and people who inject drugs (PWID) or share needles (92.0%). Statistically significant differences were observed for some risk groups. They included sex partners of people with HBV (health workers 96% vs patients 85.5%, p=0.019) and multiple sexual partners (health workers 95.2% vs patients 83.9%, p=0.04), health workers showed better awareness, as expected.

Regarding disease severity and worsening disease course, most participants agreed that viral hepatitis is deadly and requires attention (95.5%), and that co-infection worsens the disease condition (89.5%) and may affect disease management (78.4%), with statistically significant differences across all participant groups (p < 0.001). Expectedly, health workers showed higher awareness, while, interestingly, carers showed lower awareness than health workers, but consistently higher awareness than the patients themselves (**Supplementary Table 1**).

Only 24.1% agreed that not all chronic HBV cases need treatment (p<0.001), and 63.5% agreed that some infected individuals develop chronic hepatitis (p=0.029), with lower proportions of patients agreeing.

We tested for potential differences in knowledge among health care workers across different care settings (Health Centres vs Referral Hospitals). No statistically significant difference in knowledge was found for the majority of the knowledge items examined (27 out of 33 items). A trend towards greater diagnostic precision was noted in Referral Hospitals, namely, higher recognition of fatigue, fever, and unexplained weight loss as potential symptoms of viral hepatitis. Interestingly, health care participants from Health Centres seemed to have a higher recognition of two of the high-risk groups (household close contact HBV and healthcare workers exposed to blood) and a higher understanding of the fact that not all hepatitis B requires treatment. Regarding the latter, only 23% of health workers overall agreed, possibly because most of the participants in health care were nurses, who are not directly involved in treatment decisions (**Supplementary Table 2**).

### 3.2 Attitudes and practices: vaccination, healthcare and treatment preferences

Participants self-reported their vaccination history, which differed significantly across participant groups. Out of 84.9% (n = 381) of study participants who indicated having received vaccination, HWCs had the highest rate (90.6%), while patients had the lowest rate (59.7%) (p<0.001). Reported receipt of vaccination through mass vaccination campaigns was higher among HWCs (38.2%) than caregivers (17.5%) or patients (14.5%) (p<0.001), as presented in **Supplementary Table 3**.

While reported Hepatitis B vaccination was high (85.1%) and did not differ significantly between study participant groups, perceived vaccine effectiveness was strikingly low, namely only 9,7% among patients, 24,6% among carers, and just 40,3% among health workers (p<0.001). Testing for reasons for vaccine hesitancy was outside the scope of this study, but misinformation and safety concerns may be important drivers.

Regarding treatment preferences, 70.6% agreed that orthodox treatment can treat or be used to manage viral hepatitis, but 37.4% also agreed that herbal medicine can treat or manage viral hepatitis. When asked which option “improves treatment or management outcomes,” the herbal approach was chosen as best treatment mostly by patients (33.9% vs 22.8% by carers and 17.3% by health workers, p=0.004). Overall, orthodox care was deemed the best treatment by the majority of patients (54.8%), carers (68.4%), and health workers (61.2%), although the differences among groups reached the level of statistical significance (p=0.020). A smaller but important proportion (16.5%) endorsed traditional spiritual divination as the best option for managing viral hepatitis, with the highest agreement among patients (29.0%; p = 0.039).

### 3.3 Barriers to accessing hospital-based care

Compared to HWCs, patients, followed by caregivers, were significantly more concerned with the cost of medication, laboratory testing, medical consultations, and travel to the clinic, a barrier which seems to be relatively underestimated by health care providers (83.9%, p = 0.045), as shown in **Supplementary Figure S1**.

## 4 Discussion

With the WHO goal of eliminating viral hepatitis by the year 2030, there is an urgent global need to increase prevention, diagnosis, and treatment strategies of HBV infection. Understanding the disease conditions and their social impact is important, as this influences the impact of interventions. To date, there is very limited information available on the knowledge gaps that persist, the barriers to health-seeking behaviours and the beliefs regarding viral hepatitis infection in Sub-Saharan Africa.

The findings of this study reveal an important link between viral hepatitis disease condition, lived realities, and the perceptions of at-risk communities and healthcare providers in a high-burden setting in Ghana. The high general awareness but poor specific knowledge, particularly regarding Hepatitis C and D, is similar to findings from studies across several African countries, underscoring a region-wide focus on HBV that may inadvertently marginalise other hepatotoxic viruses such as HCV and HDV (Adoba et al., 2015; Mtengezo et al., 2016; Mudji et al., 2021; Vo-Quang et al., 2023; Tantuoyir et al., 2024; Abinew et al., 2025).

The disparity in serostatus awareness among HWCs (83%), caregivers (67%), and patients (45%) was alarming, as it indicates potential challenges in the integration of testing into primary care and also suggests possible psychosocial barriers that prevent individuals from seeking testing, a phenomenon documented across sub-Saharan Africa (Ncitakalo et al., 2021; Xaba et al., 2025). Study participant groups had comparatively good knowledge of symptoms, except for technical or complex ones, for which health workers consistently showed significantly better awareness.

The misconceptions regarding transmission, most notably the belief in spread through intake of large or oily meals, ageing, malaria infection, heavy manual labour or working under the sun, and most importantly, contracting viral hepatitis through witchcraft and curses, represent a fundamental barrier to effective prevention and stigma reduction (Moshabela et al., 2017; Ngere et al., 2022).

These beliefs, which hinder viral hepatitis control strategies, have been identified as drivers of social isolation and stigma for diagnosed individuals and can additionally promote family disharmony and lack of support (Gajurel and Deresinski, 2021; Ngere et al., 2022). An additional effect is experienced as it further shapes health-seeking behaviours. Attributing the cause of viral hepatitis to spirits and witchcraft highlights the need to consider dual explanatory models in awareness campaigns and education. Public health education would require acknowledgement and respectfully engaging with cultural beliefs whilst guiding them towards evidence-based disease management. Rather than dismissing them outright, a strategy which has been successfully utilised in HIV communication campaigns (Ntshebe et al., 2006; Airhihenbuwa et al., 2014).

Knowledge of hepatitis B and C symptoms and transmission routes is crucial in controlling and managing these diseases, particularly in Africa, where high prevalence is complicated by significant knowledge gaps (Lakoh et al., 2021; Maamor et al., 2022). Awareness directly influences preventive behaviours, such as HBV vaccination and safe medical practices, as observed in the current study, thereby reducing transmission. A study on viruses in Nigeria found that poor knowledge of disease transmission routes was associated with higher risk perceptions and stigmatisation (Ukaegbu et al., 2022), which may hinder disease testing and disclosure. On the contrary, improved community knowledge has been shown to increase demand for vaccination and voluntary testing, facilitating earlier diagnosis (Li et al., 2025). Even though our study showed high proportions of good knowledge of hepatitis B and C transmission routes, some participants, including health workers, indicated transmission through eating unhealthy foods, using shared spaces, and insect bites. Additionally, more than 30% of the participants were unable to recognise that some infected individuals may be asymptomatic, and this included 56.5% of the already diagnosed patients. Similar studies show inadequate knowledge in understanding asymptomatic disease conditions, mother-to-child transmission, and unsafe injections across several African countries (Guariguata et al., 2022). This gap needs to be addressed, as individuals who are unaware that they are asymptomatic carriers may unknowingly transmit the virus.

Awareness of high-risk groups for viral hepatitis can serve as a tool for prevention. The current study demonstrated that all participants had a good understanding of individuals at high risk of contracting viral hepatitis. Education campaigns can empower communities to adopt protective behaviours, address vaccine hesitancy and enhance confidence in vaccines, encourage at-risk individuals to seek vaccination (for hepatitis B) or testing, and reduce transmission by addressing stigma (Green et al., 2025). Education to identify high-risk groups requires careful community engagement, as in the midst of deep cultural beliefs, this may rather promote stigma and discrimination, which will become a barrier to essential health services.

Research in Ghana and Ethiopia identified the challenge of individuals, particularly PWID and those with chronic infections, being deterred from accessing care and treatment due to social judgment and lack of confidentiality (Adjei et al., 2019; Guure et al., 2024).

Referral hospital staff showed greater accuracy in identifying hepatitis symptoms, likely due to stronger clinical exposure and diagnostic training. However, more health centre workers were better at recognising high-risk groups and disease severity, possibly reflecting their greater involvement in public health programs that emphasise screening and referral protocols. This pattern suggests role-based differences in knowledge, where hospitals focus on diagnosis, while primary care prioritises prevention, risk identification, and early detection through guideline-driven approaches.

Most of our study participants understood the possibility that the disease condition could become severe. However, most, including some health workers (32%), were unaware of the chronic nature of the disease condition. Knowledge directly impacts the care, as demonstrated by a study in Palestine, patients with a clearer understanding of HCV treatment were more likely to complete therapy (Sawalmeh et al., 2025). Therefore, sustained, culturally adapted education campaigns that cover general awareness and specific details are essential. Such efforts enhance healthcare-seeking behaviour for testing and improve adherence to management protocols, ultimately contributing to the WHO’s elimination goals in the African region.

The study identified key barriers to assessing healthcare. Various challenges included the high cost of prescribed medications, medical investigations, the proximity of available healthcare facilities, extended hospital or clinic queues, and the availability of over-the-counter medications to manage symptoms. Ghana is a developing country, like other countries in the Sub-Saharan Africa Region, with limited healthcare facilities, infrastructure, and personnel. Thus, the capacity to meet the growing health needs of individuals is greatly limited. These challenges tend to affect the health-seeking attitudes of infected patients (Rennert et al., 2024; Nature Editorial, 2025). The implications, complex with traditional beliefs, promote seeking alternative healthcare options. This is evidenced in the current study, as a higher proportion of patients and caregivers agreed to the management of viral hepatitis disease condition at home, with herbal medication and traditional spiritual divination providing better disease management options.

The study’s strength lies in its multi-perspective design, which captures the views of patients, caregivers, and healthcare providers. However, its limitations may include its confinement to hospital settings, possibly missing the views of community members who are entirely independent from formal care, as well as the relatively limited number of patient participants, compared to health care workers.

Our results strongly support the need for a varied approach in education and the management of perceptions about viral hepatitis. This includes public health campaigns with an integrated cultural background to correct specific misconceptions and promote testing. Additionally, hepatitis education should be integrated into broader health literacy programs, particularly through school health education and mother-child clinics, to ensure early awareness and prevention throughout the life course. HWCs require ongoing professional development in patient-centred education and counselling. Finally, and most importantly, policy must address the structural barriers through sustainable financing for vaccination, decentralised testing and treatment, and the integration of hepatitis care into primary health care and existing HIV platforms to improve accessibility and efficiency.

## 5 Conclusion

This study reveals that efforts to eliminate viral hepatitis in Ghana are hindered by more than just resource constraints; widespread misconceptions also complicate them. Varied knowledge gaps, such as the comparatively low community risk perception, disease transmission myths, cost, and other practical barriers, consequently lead to late diagnosis and poor outcomes. To achieve the WHO 2030 elimination targets, it is necessary to implement integrated interventions, including destigmatising through public education, strengthening the healthcare system’s capacity for effective service delivery, and enhancing collaboration between the orthodox and traditional medicine sectors.

## Data Availability

The original contributions presented in the study are included in the article/**Supplementary Material**. Further inquiries can be directed to the corresponding author.
