# Supplementary material for: A multi-perspective assessment of knowledge, attitudes, and barriers to viral hepatitis care in Ghana
Source: Front Cell Infect Microbiol. 2026 May 13;16:1776176. doi: 10.3389/fcimb.2026.1776176 (PMC13244871; doi:10.3389/fcimb.2026.1776176)
Supplement: Supplementary file 1 [file Table1.docx]

**Supplementary Table S1: Awareness and Knowledge of Viral Hepatitis among study participants**

|  | Healthcare workers N (%) | Caregivers N (%) | Patients N (%) | Total (%) | p-value |
| --- | --- | --- | --- | --- | --- |
| Awareness & Self-reported status |  |  |  |  |  |
| Aware of Hepatitis B | 326 (98.8%) | 55 (96.5%) | 52 (83.9%) | 433 (96.4%) | <0.001* |
| Aware of Hepatitis C | 313 (94.8%) | 44 (77.2%) | 31 (50.0%) | 388 (86.4%) | <0.001* |
| Aware of Hepatitis D | 281 (85.2%) | 35 (61.4%) | 18 (29.0%) | 334 (74.4%) | <0.001* |
| Known hepatitis B/C/D status | 273 (82.7%) | 38 (66.7%) | 28 (45.2%) | 339 (75.5%) | <0.001* |
| Sources / Causes of viral hepatitis (Perceptions) |  |  |  |  |  |
| Malaria | 30 (9.1%) | 4 (7.0%) | 6 (9.7%) | 40 (8.9%) | 0.856 |
| Eating oily foods | 30 (9.1%) | 4 (7.0%) | 6 (9.7%) | 40 (8.9%) | 0.856 |
| Work under sun | 43 (13.0%) | 5 (8.8%) | 5 (8.1%) | 53 (11.8%) | 0.404 |
| Insect bites | 28 (8.5%) | 6 (10.5%) | 6 (9.7%) | 40 (8.9%) | 0.860 |
| Blood transfusion | 271 (82.1%) | 51 (89.5%) | 49 (79.0%) | 371 (82.6%) | 0.290 |
| Alcohol intake | 184 (55.8%) | 23 (40.4%) | 22 (35.5%) | 229 (51.0%) | 0.003* |
| Witchcraft/curses | 11 (3.3%) | 4 (7.0%) | 7 (11.3%) | 22 (4.9%) | 0.022* |
| Laborious work | 33 (10.0%) | 10 (17.5%) | 4 (6.5%) | 47 (10.5%) | 0.123 |
| Ageing | 67 (20.3%) | 11 (19.3%) | 11 (17.7%) | 89 (19.8%) | 0.893 |
| High food intake | 12 (3.6%) | 4 (7.0%) | 3 (4.8%) | 19 (4.2%) | 0.424 |
| Symptoms of viral hepatitis (Knowledge) |  |  |  |  |  |
| Fatigue | 286 (86.7%) | 49 (86.0%) | 47 (75.8%) | 382 (85.1%) | 0.087 |
| Fever | 266 (80.6%) | 48 (84.2%) | 43 (69.4%) | 357 (79.5%) | 0.085 |
| Chills | 211 (63.9%) | 46 (80.7%) | 30 (48.4%) | 287 (63.9%) | 0.001* |
| Shivering | 163 (49.4%) | 34 (59.6%) | 26 (41.9%) | 223 (49.7%) | 0.152 |
| Dizziness | 205 (62.1%) | 40 (70.2%) | 39 (62.9%) | 284 (63.3%) | 0.507 |
| Body weakness | 251 (76.1%) | 49 (86.0%) | 44 (71.0%) | 344 (76.6%) | 0.139 |
| Nausea/vomiting | 206 (62.4%) | 44 (77.2%) | 35 (56.5%) | 285 (63.5%) | 0.047* |
| Appetite loss | 247 (74.8%) | 45 (78.9%) | 44 (71.0%) | 336 (74.8%) | 0.605 |
| Anaemia | 217 (65.8%) | 37 (64.9%) | 26 (41.9%) | 280 (62.4%) | 0.002* |
| Diarrhoea | 142 (43.0%) | 27 (47.4%) | 22 (35.5%) | 191 (42.5%) | 0.399 |
| Abdominal pain | 238 (72.1%) | 44 (77.2%) | 41 (66.1%) | 323 (71.9%) | 0.402 |
| Dark urine | 260 (78.8%) | 44 (77.2%) | 33 (53.2%) | 337 (75.1%) | <0.001* |
| Pale stool | 216 (65.5%) | 37 (64.9%) | 26 (41.9%) | 279 (62.1%) | 0.002* |
| Jaundice | 258 (78.2%) | 46 (80.7%) | 43 (69.4%) | 347 (77.3%) | 0.253 |
| Body itch | 165 (50.0%) | 37 (64.9%) | 29 (46.8%) | 231 (51.4%) | 0.084 |
| Weight loss | 260 (78.8%) | 51 (89.5%) | 49 (79.0%) | 360 (80.2%) | 0.169 |
| Chest/abdominal pain | 255 (77.3%) | 45 (78.9%) | 46 (74.2%) | 346 (77.1%) | 0.814 |
| Liver cirrhosis | 247 (74.8%) | 47 (82.5%) | 45 (72.6%) | 339 (75.5%) | 0.396 |
| No symptom | 219 (66.4%) | 39 (68.4%) | 35 (56.5%) | 293 (65.3%) | 0.279 |
| Transmission of viral hepatitis (Knowledge) |  |  |  |  |  |
| Insect bite | 50 (15.2%) | 7 (12.3%) | 15 (24.2%) | 72 (16.0%) | 0.146 |
| Shared space | 77 (23.3%) | 12 (21.1%) | 17 (27.4%) | 106 (23.6%) | 0.698 |
| Faeco-oral | 236 (71.5%) | 29 (50.9%) | 31 (50.0%) | 296 (65.9%) | <0.001* |
| Close contact with infected person | 250 (75.8%) | 44 (77.2%) | 48 (77.4%) | 342 (76.2%) | 0.943 |
| Birth | 298 (90.3%) | 52 (91.2%) | 45 (72.6%) | 395 (88.0%) | <0.001* |
| Breastfeeding | 272 (82.4%) | 45 (78.9%) | 41 (66.1%) | 358 (79.7%) | 0.014* |
| Needle prick | 307 (93.0%) | 52 (91.2%) | 55 (88.7%) | 414 (92.2%) | 0.50 |
| Blood transfusion | 311 (94.2%) | 55 (96.5%) | 55 (88.7%) | 421 (93.8%) | 0.173 |
| Tattooing | 279 (84.5%) | 50 (87.7%) | 48 (77.4%) | 377 (84.0%) | 0.266 |
| Sharing personal items | 306 (92.7%) | 55 (96.5%) | 55 (88.7%) | 416 (92.7%) | 0.267 |
| Sexual intercourse | 306 (92.7%) | 54 (94.7%) | 51 (82.3%) | 411 (91.5%) | 0.011* |
| Unhealthy foods | 125 (37.9%) | 12 (21.1%) | 12 (19.4%) | 149 (33.2%) | 0.002* |
| High-risk groups for viral hepatitis (Knowledge) | |  |  |  |  |
| Sex partners of people with HBV | 317 (96.1%) | 52 (91.2%) | 53 (85.5%) | 422 (94.0%) | 0.019* |
| People who inject drugs or share needles equipment | 306 (92.7%) | 53 (93.0%) | 54 (87.1%) | 413 (92.0%) | 0.305 |
| Infants born to HBV mothers | 305 (92.4%) | 49 (86.0%) | 52 (83.9%) | 406 (90.4%) | 0.061 |
| Household close contact HBV | 218 (66.1%) | 41 (71.9%) | 45 (72.6%) | 304 (67.7%) | 0.227 |
| Healthcare workers exposed to blood | 296 (89.7%) | 50 (87.7%) | 49 (79.0%) | 395 (88.0%) | 0.050 |
| Dialysis patients | 213 (64.5%) | 35 (61.4%) | 38 (61.3%) | 286 (63.7%) | 0.275 |
| Multiple sex partners | 314 (95.2%) | 53 (93.0%) | 52 (83.9%) | 419 (93.3%) | 0.040* |
| Viral hepatitis disease severity (Knowledge) |  |  |  |  |  |
| Hepatitis deadly needs attention | 319 (96.7%) | 55 (96.5%) | 55 (88.7%) | 429 (95.5%) | <0.001* |
| Coinfection worsens disease status | 296 (89.7%) | 52 (91.2%) | 54 (87.1%) | 402 (89.5%) | <0.001* |
| Coinfection affects disease management | 265 (80.3%) | 45 (78.9%) | 42 (67.7%) | 352 (78.4%) | <0.001* |
| Not all chronic HBV need treatment | 76 (23.0%) | 16 (28.1%) | 16 (25.8%) | 108 (24.1%) | <0.001 |
| Some infected develop chronic hepatitis | 224 (67.9%) | 31 (54.4%) | 30 (48.4%) | 285 (63.5%) | 0.029* |
| Notes: Values are Agree/Strongly Agree N (%) within each group. For items coded Yes/No, Yes is treated as agreement. | | | | | |
| P-values: Compare response distributions across groups using Pearson Chi-square test. If any expected cell count < 5, Fisher’s exact test was used for 2×2 tables; otherwise, Chi-square with simulated p-value (Monte Carlo, B = 2000). * p-values<0.05 | | | | | |
