# Supplementary material for: A multi-perspective assessment of knowledge, attitudes, and barriers to viral hepatitis care in Ghana
Source: Front Cell Infect Microbiol. 2026 May 13;16:1776176. doi: 10.3389/fcimb.2026.1776176 (PMC13244871; doi:10.3389/fcimb.2026.1776176)
Supplement: Supplementary file 2 [file Table2.docx]

**Supplementary Table S2.**

Awareness and knowledge of viral hepatitis among health professionals in different level of health facility type setting

| Variable | Health Centre Settings n = 34, N (%) | Referral Hospital Setting n = 293, N (%) | Total N (%) | p-value |
| --- | --- | --- | --- | --- |
| Awareness & Self-reported status |  |  |  |  |
| Known hepatitis B/C/D status | 29 (85.3%) | 251 (85.7%) | 280 (85.6%) | 1.000 |
| Sources / Causes of viral hepatitis (Perceptions) |  |  |  |  |
| Blood transfusion | 26 (76.5%) | 242 (82.6%) | 268 (82.0%) | 0.379 |
| Alcohol intake | 18 (52.9%) | 163 (55.6%) | 181 (55.4%) | 0.765 |
| Witchcraft/curses | 0 (0.0%) | 10 (3.4%) | 10 (3.1%) | 0.607 |
| Ageing | 3 (8.8%) | 64 (21.8%) | 67 (20.5%) | 0.075 |
| Symptoms of viral hepatitis (Knowledge) |  |  |  |  |
| Fatigue | 24 (70.6%) | 259 (88.4%) | 283 (86.5%) | **0.013*** |
| Fever | 19 (55.9%) | 245 (83.6%) | 264 (80.7%) | **<0.001*** |
| Abdominal pain | 21 (61.8%) | 214 (73.0%) | 235 (71.9%) | 0.166 |
| Dark urine | 23 (67.6%) | 234 (79.9%) | 257 (78.6%) | 0.100 |
| Pale stool | 18 (52.9%) | 196 (66.9%) | 214 (65.4%) | 0.105 |
| Jaundice | 23 (67.6%) | 232 (79.2%) | 255 (78.0%) | 0.124 |
| Weight loss | 22 (64.7%) | 236 (80.5%) | 258 (78.9%) | **0.032*** |
| Liver cirrhosis | 24 (70.6%) | 220 (75.1%) | 244 (74.6%) | 0.568 |
| No symptom | 22 (64.7%) | 196 (66.9%) | 218 (66.7%) | 0.798 |
| Transmission of viral hepatitis (Knowledge) |  |  |  |  |
| Insect bite | 7 (20.6%) | 43 (14.7%) | 50 (15.3%) | 0.365 |
| Shared space | 8 (23.5%) | 68 (23.2%) | 76 (23.2%) | 0.967 |
| Birth | 33 (97.1%) | 263 (89.8%) | 296 (90.5%) | 0.226 |
| Needle prick | 33 (97.1%) | 271 (92.5%) | 304 (93.0%) | 0.489 |
| Blood transfusion | 34 (100.0%) | 274 (93.5%) | 308 (94.2%) | 0.239 |
| Tattooing | 32 (94.1%) | 244 (83.3%) | 276 (84.4%) | 0.099 |
| Sexual intercourse | 33 (97.1%) | 271 (92.5%) | 304 (93.0%) | 0.489 |
| High-risk groups for viral hepatitis (Knowledge) |  |  |  |  |
| Sex partners of people with HBV | 34 (100.0%) | 280 (95.6%) | 314 (96.0%) | 0.376 |
| People who inject drugs or share needle equipment | 33 (97.1%) | 270 (92.2%) | 303 (92.7%) | 0.490 |
| Infants born to HBV mothers | 31 (91.2%) | 271 (92.5%) | 302 (92.4%) | 0.734 |
| Household close contact HBV | 30 (88.2%) | 185 (63.1%) | 215 (65.7%) | **0.004*** |
| Healthcare workers exposed to blood | 34 (100.0%) | 259 (88.4%) | 293 (89.6%) | **0.034*** |
| Dialysis patients | 27 (79.4%) | 183 (62.5%) | 210 (64.2%) | 0.051 |
| Multiple sex partners | 34 (100.0%) | 277 (94.5%) | 311 (95.1%) | 0.390 |
| Viral hepatitis disease severity (Knowledge) |  |  |  |  |
| Hepatitis deadly needs attention | 34 (100.0%) | 282 (96.2%) | 316 (96.6%) | 0.613 |
| Coinfection worsens disease status | 31 (91.2%) | 262 (89.4%) | 293 (89.6%) | 1.000 |
| Coinfection affects disease management | 27 (79.4%) | 236 (80.5%) | 263 (80.4%) | 0.875 |
| Not all chronic HBV need treatment | 13 (38.2%) | 63 (21.5%) | 76 (23.2%) | **0.029*** |
| Some infected develop chronic hepatitis | 26 (76.5%) | 196 (66.9%) | 222 (67.9%) | 0.258 |
| Notes: Values are Agree/Strongly Agree N (%) within each group. For items coded Yes/No, Yes is treated as agreement  Three healthcare professionals with missing setting type were excluded. | | | | |
| Response distributions were compared between Health centre settings and Referral Hospital settings. Pearson's chi-square test was used when expected cell counts were adequate; Fisher’s exact test was used when expected cell counts were < 5. * p < 0.05. | | | | |
