# Supplementary material for: A multi-perspective assessment of knowledge, attitudes, and barriers to viral hepatitis care in Ghana
Source: Front Cell Infect Microbiol. 2026 May 13;16:1776176. doi: 10.3389/fcimb.2026.1776176 (PMC13244871; doi:10.3389/fcimb.2026.1776176)
Supplement: Supplementary file 3 [file Table3.docx]

**Supplementary Table S3: Attitudes and Practices of study participants (Vaccination, Healthcare and Treatment Preferences)**

|  | Health workers  N (%) | Caregivers  N (%) | Patients  N (%) | Total  N (%) | p-value |
| --- | --- | --- | --- | --- | --- |
| Vaccination (Practices/Perceptions) | | | | | |
| Mass vaccination | 126 (38.2%) | 10 (17.5%) | 9 (14.5%) | 145 (32.3%) | <0.001* |
| Hep B | 284 (86.1%) | 50 (87.7%) | 48 (77.4%) | 382 (85.1%) | 0.180 |
| BCG | 158 (47.9%) | 37 (64.9%) | 37 (59.7%) | 232 (51.7%) | 0.024* |
| DPT3 | 135 (40.9%) | 29 (50.9%) | 32 (51.6%) | 196 (43.7%) | 0.148 |
| Measles | 194 (58.8%) | 42 (73.7%) | 47 (75.8%) | 283 (63.0%) | 0.008* |
| Yellow fever | 235 (71.2%) | 42 (73.7%) | 42 (67.7%) | 319 (71.0%) | 0.769 |
| Tetanus | 185 (56.1%) | 37 (64.9%) | 36 (58.1%) | 258 (57.5%) | 0.456 |
| Pentavalent | 325 (98.5%) | 57 (100.0%) | 62 (100.0%) | 444 (98.9%) | 0.469 |
| Effectiveness | 133 (40.3%) | 14 (24.6%) | 6 (9.7%) | 153 (34.1%) | <0.001* |
| Healthcare Access and Barriers | | | | | |
| Long hospital waiting time | 187 (56.7%) | 39 (68.4%) | 41 (66.1%) | 267 (59.5%) | 0.114 |
| Unpleasant health attendants’ attitude | 198 (60.0%) | 39 (68.4%) | 42 (67.7%) | 279 (62.1%) | 0.077 |
| Distance to clinic far/high transport cost | 193 (58.5%) | 37 (64.9%) | 43 (69.4%) | 273 (60.8%) | 0.034* |
| High cost of medicines and consultation | 238 (72.1%) | 47 (82.5%) | 52 (83.9%) | 337 (75.1%) | 0.045* |
| Can obtain medications from retail outlets | 124 (37.6%) | 30 (52.6%) | 29 (46.8%) | 183 (40.8%) | 0.118 |
| Diagnostic lab tests too expensive | 204 (61.8%) | 44 (77.2%) | 50 (80.6%) | 298 (66.4%) | 0.007* |
| Treatment Preferences | | | | | |
| Hepatitis can be treated at home | 57 (17.3%) | 12 (21.1%) | 18 (29.0%) | 87 (19.4%) | 0.138 |
| Herbal can treat manage hepatitis | 124 (37.6%) | 21 (36.8%) | 23 (37.1%) | 168 (37.4%) | 0.226 |
| Orthodox can treat manage hepatitis | 231 (70.0%) | 41 (71.9%) | 45 (72.6%) | 317 (70.6%) | 0.438 |
| Home treatments improve outcomes best | 52 (15.8%) | 16 (28.1%) | 17 (27.4%) | 85 (18.9%) | 0.079 |
| Herbal improve outcomes best | 57 (17.3%) | 13 (22.8%) | 21 (33.9%) | 91 (20.3%) | 0.004* |
| Orthodox improve outcomes best | 202 (61.2%) | 39 (68.4%) | 34 (54.8%) | 275 (61.2%) | 0.020* |
| Traditional spiritual divination best | 47 (14.2%) | 9 (15.8%) | 18 (29.0%) | 74 (16.5%) | 0.039* |
| Attitudes / Beliefs | | | | | |
| HBV and HCV major diseases in Ghana | 281 (85.2%) | 49 (86.0%) | 49 (79.0%) | 379 (84.4%) | 0.307 |
| Hepatitis deadly needs attention | 319 (96.7%) | 55 (96.5%) | 55 (88.7%) | 429 (95.5%) | <0.001* |
| Coinfection worsens disease status | 296 (89.7%) | 52 (91.2%) | 54 (87.1%) | 402 (89.5%) | <0.001* |
| Coinfection affects disease management | 265 (80.3%) | 45 (78.9%) | 42 (67.7%) | 352 (78.4%) | <0.001* |
| Not all chronic HBV need treatment | 76 (23.0%) | 16 (28.1%) | 16 (25.8%) | 108 (24.1%) | <0.001* |
| Hepatitis completely curable | 147 (44.5%) | 20 (35.1%) | 27 (43.5%) | 194 (43.2%) | 0.717 |
| Some infected develop chronic hepatitis | 224 (67.9%) | 31 (54.4%) | 30 (48.4%) | 285 (63.5%) | 0.029* |
| Mass vaccination: Received vaccination through a mass vaccination campaign  Pentavalent vaccine: DTP-HepB-Hib vaccine (diphtheria, tetanus, pertussis, hepatitis B, and *Haemophilus influenzae* type b) | | | | | |
| Notes: Values are Agree/Strongly Agree N (%) within each participant group (Healthcare, Caregivers, Patients). For items coded Yes/No, Yes is treated as agreement. | | | | | |
| P-values: Compare response distributions across groups using Pearson Chi-square test. If any expected cell count < 5, Fisher’s exact test was used for 2×2 tables; otherwise, Chi-square with simulated p-value (Monte Carlo, B = 2000). * p-value<0.005 | | | | | |
