# Supplementary figures and images for: A multi-perspective assessment of knowledge, attitudes, and barriers to viral hepatitis care in Ghana
Source: Front Cell Infect Microbiol. 2026 May 13;16:1776176. doi: 10.3389/fcimb.2026.1776176 (PMC13244871; doi:10.3389/fcimb.2026.1776176)

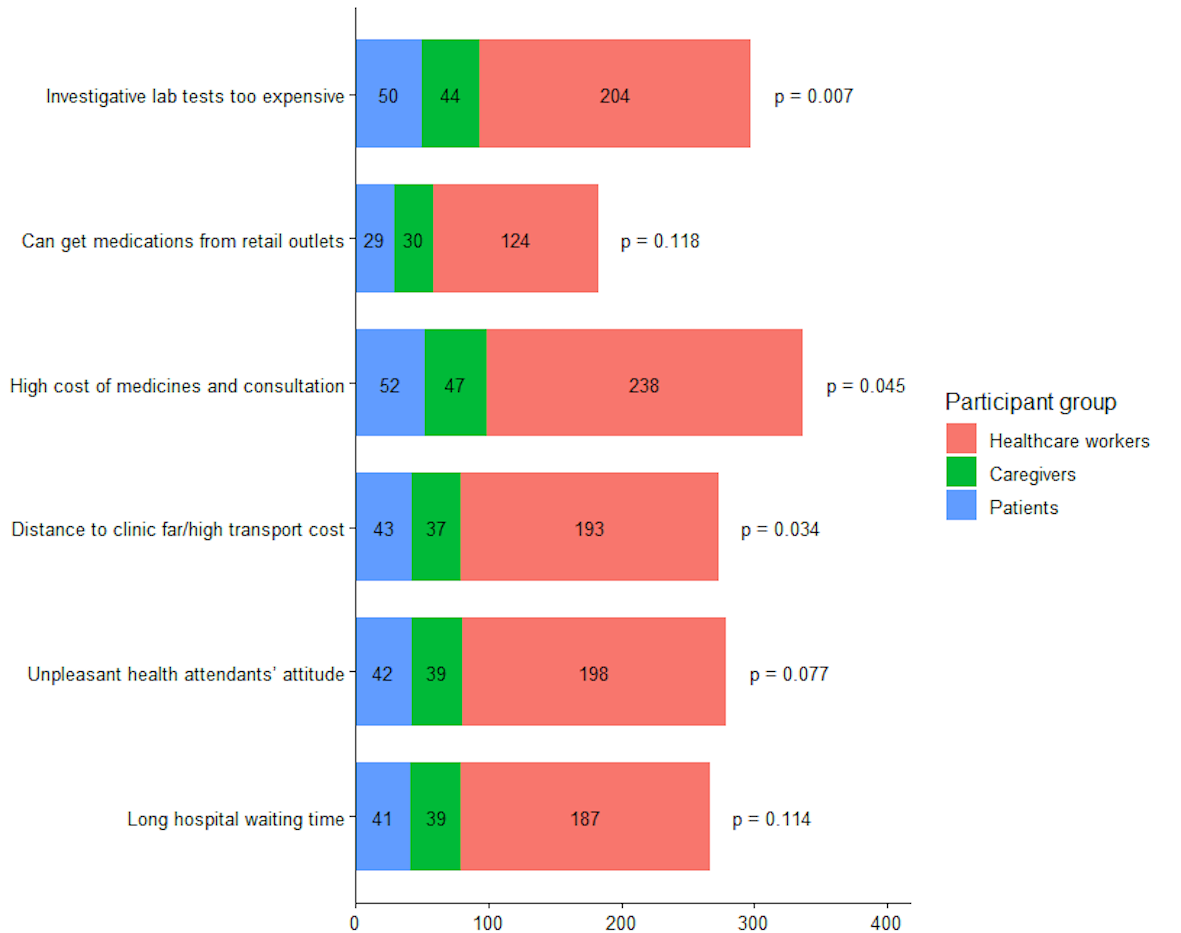

Supplement: Supplementary file 4 [file Image1.tiff]
